# Supplementary material for: A cross-sectional study of dental students perception of dental faculty gender differences
Source: PLoS One. 2022 Jul 29;17(7):e0271570. doi: 10.1371/journal.pone.0271570 (PMC9337690; doi:10.1371/journal.pone.0271570)
Supplement: S1 File — (DOCX) [file pone.0271570.s001.docx]

**Questionnaire**

**Please select the appropriate response. There is no right or wrong answer to any of the questions listed.**

1. **Which year are you studying at?**

□ Second-year dental student (D2)

□ Third-year dental student (D3)

□ Final year dental student (D4)

1. **How old are you?**

□ 20-23

□ 24-26

□ 27-30

□ Greater than 30

1. **Gender:**

□ Male

□ Female

□ Other (Please specify) ______________________

1. **Which ethnic group best describes you?**

□ Caucasian/White

□ African American/Black

□ Hispanic/Latino

□ Asian

□ Mixed/multiple ethnic groups

□ Other (Please specify) ______________________

1. **How many times per week did you work with a Female faculty?**

Stimulation clinic □ Never □ 1-2 □ More than 3

Clinic □ Never □ 1-2 □ More than 3

1. **How many times per week did you work with a Male faculty?**

Stimulation clinic □ Never □ 1-2 □ More than 3

Clinic □ Never □ 1-2 □ More than 3

1. **Please select your level of agreement with the following statements**

| **Items** | **Strongly**  **Agree** | **Agree** | **Neutral** | **Disagree** | **Strongly Disagree** |
| --- | --- | --- | --- | --- | --- |
| I prefer working with **Male** faculty in stimulation clinic |  |  |  |  |  |
| I prefer working with **Male** faculty in Clinic |  |  |  |  |  |
| I prefer working with **Female** faculty in stimulation |  |  |  |  |  |
| I prefer working with **Male** faculty in Clinic |  |  |  |  |  |
| I prefer discussing my personal problems with a **Male** Faculty |  |  |  |  |  |
| Having a diverse faculty positively affect your learning |  |  |  |  |  |

1. **On the scale of 1 to 5 (5 being the highest number), please select your level of preference for each category (Please fill in each column)**

| **Items** | **Male Faculty** | **Female Faculty** | **Not Applicable** |
| --- | --- | --- | --- |
| Prefer working with in **Restorative Dentistry** |  |  |  |
| Prefer working with in **Oral Surgery** |  |  |  |
| Prefer working with in **Pediatric Dentistry** |  |  |  |
| Prefer working with while doing **Orthodontic Procedures** |  |  |  |
| Prefer working with while doing **Endodontics Procedures** |  |  |  |
| Prefer working with while doing **Periodontal Procedures** |  |  |  |

1. **On the scale of 1 to 5 (5 being the highest score), please score faculty for each category (Please fill in each column)**

| **Items** | **Male Faculty** | **Female Faculty** | **Not Applicable** |
| --- | --- | --- | --- |
| Provides constructive feedback |  |  |  |
| More approachable |  |  |  |
| More Knowledgeable/skills |  |  |  |
| Encourages Critical thinking |  |  |  |
| Encourages discussions |  |  |  |
| Work effectively with others |  |  |  |
| Supportive |  |  |  |
| Motivational/Encouraging |  |  |  |
| Communicates well with patients |  |  |  |
| Communicates with students |  |  |  |
| Communicates with staff |  |  |  |
| Works effectively with other faculty |  |  |  |
| Is a role model |  |  |  |
| More accepting to cultural differences |  |  |  |
| Provides better understanding of cultures |  |  |  |
| trust your clinical judgment more |  |  |  |
| Gives you independence in performing procedures |  |  |  |
| Who challenges your knowledge/credibility |  |  |  |
| Who is more rigid/inflexible |  |  |  |
| Exerts superiority |  |  |  |
| Harder on grading |  |  |  |

**Thank you for completing the questionnaire**
