# Supplementary material for: A cross-sectional study of dental students perception of dental faculty gender differences
Source: PLoS One. 2022 Jul 29;17(7):e0271570. doi: 10.1371/journal.pone.0271570 (PMC9337690; doi:10.1371/journal.pone.0271570)
Supplement: Attachment — Submitted filename: Response to Reveiwers.docx [file pone.0271570.s002.docx]

Response to Reviewers

Please use the space provided to explain your answers to the questions above. You may also include additional comments for the author, including concerns about dual publication, research ethics, or publication ethics. (Please upload your review as an attachment if it exceeds 20,000 characters)

**Reviewer #1:**

**This is a very interesting study. Please note this reviewer’s comments below.**

**Title:
Regarding the title of Dental Student’s Perception of Gender Differences among Dental Faculty: A Cross- sectional Study
It might be better stated as the following since it only relates to one dental school approach or as: A Cross-sectional Study of Dental Student Perception of Dental Faculty Gender Differences**

Response: Title has been modified as suggested.

Revised title: A Cross-sectional Study of Dental Student Perception of Dental Faculty Gender Differences

**Page 3 Lines 56-57 regarding
Mentors and role models’ effect on gender related specialty preference has been well- documented in medicine. [8,9]
Please rephrase this statement for clarity.**

Response: Statements have been rephrased and sentence structure reviewed.

Revised sentence: It has been well-documented in medicine that mentors and role models can have an impact on different genders’ choices of a specific specialty over another.

**Page 4 Lines 75-76 regarding
Survey was administered separately among all clinical years in class and collected from all students regardless of participation.
Can you please explain what is meant by regardless of participation?**

Response: Due to the sensitivity of the topic. No signed consent was obtained. Filling out the survey was used as implied informed consent. The entire class was presented with the survey and was informed not to fill it out if they did not wish to participate. All surveys were collected back regardless of the participation.

**Line 77 regarding
No signed consent forms were collected to protect student privacy. Filling the survey was used as implied informed consent. Please rephrase these two sentences for clarity.**

Response: Rephrased as suggested.

Revised text: For student privacy protection, no identifiable information was included in the survey. The entire class was presented with the survey and was informed not to fill it out if they did not wish to participate. All surveys were collected back regardless of the participation. No signed consent was obtained. Filling out the survey was used as implied informed consent.

**Page 6 Table 1, is this table necessary since the content has been documented within the manuscript.**

Response: Although some of the data from Table 1 was included in the text. Having the table in manuscript will allow comparison for future studies when they compare their student population to ours.

**Page 13 Line 173 regarding
This difference highlights the importance of gender matching and having females in academic roles to inspire female students.
Do you have a reference for this sentence?**

Response: This statement was a reflection based on the results of our study and the studies reviewed in the paragraph. No citations.

**Page 14 Line 192 regarding
To increase dental faculty cultural competency, schools need to provide faculty development seminars on gender and cultural awareness.
Did you mean cultural diversity vs. cultural competency? One can never be fully culturally competent since it is a journey. The word cultural competency shows up during the entire manuscript whereas a better fit can be cultural diversity and not competency.**

Response: Cultural competency was changed to cultural diversity throughout the manuscript.

**Regarding Methodology
Can you please share the survey as a table so that others can duplicate this work?**

Response: It is hard to make the full survey as a table but it will be included as a supplemental material.

**Can you explain the high survey response rate if it was on a volunteer basis and confidential?**

Response: Yes. The survey is on a volunteer basis and confidential. We think that they high response rate was because of students interest in participation.

**Did you have a chance to compare D2, to D3 and D4 years of training regarding for any statistical differences? In other words, are the reports more significant or less significant as the dental students advance to clinical confidence?**

Response: Year of study and preference of working with faculty were compared . Following statement were added in the result section.

There was no significant difference in students preferences scores according to their age or year of study (p>0.05).

**Reviewer #2: This study (survey) is well conducted and reported. Data in table 2 (Students' exposure to faculty of different genders) may be better presented as a figure (Histogram). Data in tables 3-6 should be defined e.g. Mean (SD)**

Response: Mean are added on tables 3-6 according to suggestions.

3. Authors noted the following: "Yes - all data are fully available without restriction." "The data underlying the results

presented in the study is available on request from Shaista Rashid."

**Reviewer #3: 1. Discussion on the limitations of the manuscript should be expanded further. Comments regarding cultural/racial/ethnic differences are limited since the majority of the sample were Caucasian/White students. Lumping the non-White students together appeared to be for purely statistical analysis reasons, but can be misleading since there are likely differences in perception/experiences among African American/Black, Hispanic/Latino, Asian, and Mixed/multiple ethnic groups.**

Response: Limitation is added according to suggestion in discussion section.

In this study, all non-White students were grouped into the “Others” category due to the small size of participants from each population. There are likely differences with members of this groups in their perceptions and experiences. Given these limitations, generalizability related to all non-White students is limited

4. **The manuscript needs to be edited throughout for grammar and clarity. For example, the use of apostrophes, especially accurately discerning between singular and plural, needs to be addressed. On p. 2, please explain "assorted prospective." On p. 7, please clarify ". . . no difference between male and female students in preserving female faculty as role models." On p. 12, need to edit: "This difference could also by (sic) sex stereotyping of women as being. . ."**

Response: Manuscript has been reviewed and reformatted for clarity according to suggestion.
