# Supplementary material for: A cross-sectional study of dental students perception of dental faculty gender differences
Source: PLoS One. 2022 Jul 29;17(7):e0271570. doi: 10.1371/journal.pone.0271570 (PMC9337690; doi:10.1371/journal.pone.0271570)
Supplement: Attachment — Submitted filename: Response to Reviewers.docx [file pone.0271570.s003.docx]

**Response to Reviewers**

Please use the space provided to explain your answers to the questions above. You may also include additional comments for the author, including concerns about dual publication, research ethics, or publication ethics. (Please upload your review as an attachment if it exceeds 20,000 characters)

**Reviewer #1: The manuscript has improved with your additional edits. This reviewer has additional comments:

On page 4 regarding: Data were collected using a self-administered questionnaire adopted from the cognitive apprenticeship model in clinical practice.[18] Formatting...
Please check Formatting... and clarify the word.**

Response: Formatting was fixed and words were removed for clarification. **On page 5 regarding: All questions have a not applicable (N/A) option.
Should it be All questions included a not applicable (N/A) option.**

Response. Statement has been rephrased.

Revised sentence: All questions included a not applicable (N/A) option. **On page 13 regarding: This difference highlights the importance of gender matching and having females in academic roles to inspire female students.
Please rephrase this sentence by adding that: It to it is our belief or our reflection or that we speculate that "this difference highlights the importance of gender matching and having females in academic roles to inspire female students or something like that to highlight your reflection vs. a quotation."**

Response: Statements have been rephrased and sentence structure reviewed.

Revised sentence: We postulate that this difference highlights the importance of gender matching and having females in academic roles to inspire female students. **It would have been instrumental to read the specific survey questions; however, the questionnaire was not included. The origin of the survey should be documented as well as where the questions came from or how they were formatted.**

Response: The questionnaire is now included as a supplemental material.

**Reviewer #2: Table 4. Student’s mean (SD) perception scores for male and female faculty according to student’s gender and ethnicity
First row includes Same applied for Male Faculty and Female Faculty twice and NO students Male and Female data. or Table 6.**

Response: In both table 4 and 6, the first 2 columns represent the students mean (SD) perception of male and female faculty based on student gender (male vs female). The third and fourth columns represents mean (SD) perception of male and female faculty based on student ethnicity (white vs other)
